# Hypoxia and Hypoxia-Inducible Factors in Leukemias

**DOI:** 10.3389/fonc.2016.00041

**Published:** 2016-02-26

**Authors:** Margaux Deynoux, Nicola Sunter, Olivier Hérault, Frédéric Mazurier

**Affiliations:** ^1^Génétique, Immunothérapie, Chimie et Cancer (GICC) UMR 7292, CNRS, UFR de Médecine, Université François-Rabelais de Tours, Tours, France; ^2^Service d’Hématologie Biologique, Centre Hospitalier Régional Universitaire de Tours, Tours, France

**Keywords:** hypoxia, hypoxia-inducible factors, leukemia, microenvironment, cancer

## Abstract

Despite huge improvements in the treatment of leukemia, the percentage of patients suffering relapse still remains significant. Relapse most often results from a small number of leukemic stem cells (LSCs) within the bone marrow, which are able to self-renew, and therefore reestablish the full tumor. The marrow microenvironment contributes considerably in supporting the protection and development of leukemic cells. LSCs share specific niches with normal hematopoietic stem cells with the niche itself being composed of a variety of cell types, including mesenchymal stem/stromal cells, bone cells, immune cells, neuronal cells, and vascular cells. A hallmark of the hematopoietic niche is low oxygen partial pressure, indeed this hypoxia is necessary for the long-term maintenance of hematopoietic stem/progenitor cells. Hypoxia is a strong signal, principally maintained by members of the hypoxia-inducible factor (HIF) family. In solid tumors, it has been well established that hypoxia triggers intrinsic metabolic changes and microenvironmental modifications, such as the stimulation of angiogenesis, through activation of HIFs. As leukemia is not considered a “solid” tumor, the role of oxygen in the disease was presumed to be inconsequential and remained long overlooked. This view has now been revised since hypoxia has been shown to influence leukemic cell proliferation, differentiation, and resistance to chemotherapy. However, the role of HIF proteins remains controversial with HIFs being considered as either oncogenes or tumor suppressor genes, depending on the study and model. The purpose of this review is to highlight our knowledge of hypoxia and HIFs in leukemic development and therapeutic resistance and to discuss the recent hypoxia-based strategies proposed to eradicate leukemias.

## Leukemias and Hypoxia

Leukemia is characterized by uncontrolled proliferation of hematopoietic cells within bone marrow (BM). Lymphoid leukemias can be distinguished from myeloid according to the abnormal cell lineage, and acute from chronic leukemias according to the maturity of the blood cells involved and progression rate. Acute leukemias are characterized by rapid proliferation of immature hematopoietic cells, termed blasts, which fail to differentiate into mature cells. Their accumulation in BM also prevents growth and differentiation of normal hematopoietic cells. The clinical evolution is fast (1). In contrast, in chronic leukemia, the growth advantage of neoplastic cells leads to the generation of a more mature cell population that outcompetes normal hematopoiesis, and clinical evolution is longer (several months to years) (2). To date, although the majority of pediatric acute lymphoid leukemia (ALL) and chronic myeloid leukemia (CML) cases (3) are cured or well controlled under treatment, chronic lymphoid leukemia (CLL) and, even more, acute myeloid leukemia (AML) have a high risk of relapse, despite therapeutic progressions (4). While treatments often target cycling cells, the idea that a small population of quiescent leukemic cells survive and trigger relapse regardless of treatment has emerged (5). In the early 90s, the team of John E. Dick established a hierarchy in leukemic cell populations which, by analogy with that of normal hematopoietic cells, led to the introduction of the concept of cancer stem cells for all cancers [reviewied in Ref. (5, 6)]. Their work identified a subpopulation of leukemic cells able to initiate leukemic growth after transplantation into immune-deficient mice. These stem-like cells, named leukemia-initiating cells (LICs) or leukemic stem cells (LSCs) (7, 8), arise from hematopoietic stem/progenitor cells (HSCs) that reside in the most hypoxic tissue areas within the normal HSC niche (9, 10). The oxygen partial pressure (ppO_2_) in tissues is much lower compared to that in the atmosphere (160 mmHg corresponding to around 21% oxygen). In particular, in BM an oxygen gradient exists ranging from <6% oxygen, close to the vessels, to anoxia in the most distant regions from blood vessels (11–15). However, O_2_ level differs according to the nature of the hematopoietic niche; the sinusoidal niche is around 10 mmHg (equivalent to 1.3% O_2_) (16).

In solid cancers, it is well established that uncontrolled proliferation leads to profound hypoxia, associated with tumor development, metabolic changes, metastatic propagation, immune response modulation, and increased mortality (17, 18). Consequently, it could be assumed that intense blast cell proliferation would eventually decrease the oxygen availability by high consumption. This assumption is particularly difficult to validate by direct measurement in human BM. However, Fiegl et al. (19) demonstrated in total BM aspirates from AML patients that oxygen percentage was highly comparable to the normal counterpart. Using a rat model of an acute AML subtype, the promyelocytic leukemia, Jensen et al. (20) noted an increasing level of hypoxia during disease progression, comparable to that observed with solid tumours. In this model, both normal and leukemic cells stained with 2-nitroimidazole (hypoxic marker) underwent decreased proliferation. In agreement with this observation, the hypoxic culture of normal hematopoietic (21–23) and CML cells (24–27) led to decreased proliferation. Nevertheless, a small fraction of leukemic cells remained insensitive to hypoxia-induced proliferation arrest (26), probably triggering tumor growth (20). Moreover, it has been established that mild hypoxia such as ≤3% O_2_ sustains both primary CML (24) and AML cell (28) maintenance longer than normoxia.

Low oxygen might also affect hematopoietic cells through the modulation of the stromal cells. Indeed, hypoxia has been shown to impact on survival, proliferation capability, and differentiation as well as metabolism of mesenchymal stem/stromal cells (MSCs) (29–31). Hypoxia triggers secretion by MSCs of numerous factors, including SDF-1, VEGF, and IL-6, known to promote HSC maintenance. Interestingly, even in normoxia, HSCs present a hypoxic profile when seeded on MSCs, suggesting appearance of “microhypoxic” regions (32, 33). Moreover, MSCs and hypoxic culture synergize to sustain *in vitro* normal stem cells (23) and primary AML cells (28). Finally, the poorly oxygenized niche enhances resistance to treatments (28, 34, 35), thus protecting from various stresses, such as DNA damage, cell death stimuli, or oxidative stress signals (36–38).

## Hypoxia-Inducible Factors in Leukemias

The master regulators mediating cell responses to hypoxia are the hypoxia-inducible factors (HIFs). These heterodimer complexes are composed of one of three oxygen-regulated HIF-alpha subunits (HIF-1alpha, HIF-2alpha, and HIF-3alpha) and the constitutively expressed HIF-beta subunit [HIF-1-beta, also known as aryl hydrocarbon receptor nuclear translocator (ARNT)] (39–41). The *HIF1A* gene is ubiquitously expressed (42). *HIF2A*, also termed endothelial Per-ARNT-Sim (PAS) protein 1 (*EPAS1*), is expressed in a more tissue specific manner, particularly in blood vessels (39, 40, 43). Little is currently known about expression and function of *HIF3A*, but at least 10 splice variants have been described to date (44, 45). HIF-1alpha and HIF-2alpha proteins share similar structural domains such as an N-terminal basic helix-loop-helix (bHLH) domain involved in DNA binding, the two PAS domains allowing dimerization, an oxygen-dependent degradation domain (ODDD) plus N- and C-terminal transactivation domains (NTAD and CTAD). Although HIF-3alpha also exhibits high similarity in bHLH and PAS domains, the lack of the CTAD precludes binding to p300 coactivator (45, 46). Under atmospheric conditions, HIF-alpha subunits are differentially hydroxylated by prolyl hydroxylase domain 1–3 (PHDs) on two proline residues in the ODDD, with oxygen and α-ketoglutarate as substrates. The hydroxylated motif allows binding to von Hippel–Lindau (VHL) tumor suppressor, which leads to HIF-alpha ubiquitination and consequent degradation by 26S proteasome (39–41, 47). In parallel, the hydroxylation of HIF-1alpha by factor inhibiting HIF-1 (FIH1) triggers inhibition of p300/CBP coactivator recruitment (48). PHD activity falls with decreasing oxygen levels, thereby triggering HIF-alpha stabilization and nuclear translocation where it heterodimerizes with HIF-1beta. HIF complexes bind to specific HIF-response elements consisting of specific RCGTG sequences within target gene promoters. Although HIF-1 and HIF-2 share common targets, additional genomic regions and cofactor-binding specifically drive the transcriptional initiation of genes involved in many pathways, such as angiogenesis, differentiation, stem cells maintenance, apoptosis, and invasion (35, 39, 40).

HIF-1alpha mainly participates in the initial response to acute hypoxia, whereas HIF-2alpha responds to chronic exposure (47, 49). Additionally, even in prolonged hypoxia HIF-1alpha undergoes feedback control, whereas HIF-2alpha is stabilized. The multiple HIF-3alpha splice variants appear essentially to regulate HIF-1alpha and HIF-2alpha activity by sequestrating HIF-1beta or by acting as dominant negative regulators (40, 44, 45, 50). HIFs, moreover, can be regulated by oxygen-independent mechanisms. Factors involved in hematopoiesis such as MEIS1 (51), TPO (52), and SCF (53, 54) positively regulate expression of HIFs. Conversely, factors implicated in metabolic changes like the SIRT1 (55, 56) or SDH (57) inhibit the expression of HIFs. Furthermore, downregulation of HIFs may be achieved by tumor suppressor genes like p53 (58, 59) or GSK3 (60) and the upregulation by oncogenes such as PI3K/AKT (60, 61) or mTORC1 (62). Genetic abnormalities encountered in leukemia such as the *IDH* mutation decrease expression of HIFs by stimulating PHD activity (63) or conversely, *FLT3-ITD* stimulates translation of HIFs *via* the PI3K/AKT pathway (40).

Elevated expression of HIFs is considered to be a marker of poor prognosis in solid cancers (64–77). Overall, increased expression of HIFs is correlated with tumor growth and resistance to therapies, which leads to disease relapse (37). The subject is somewhat more complex and controversial in leukemia. Overexpression of HIF-1alpha in leukemia has been suggested as a marker of poor prognosis and chemotherapy outcomes (78–81). Elevated levels of HIF-1alpha are reported, in the majority of studies, in AML (79, 82–84), acute promyelocytic leukemia (APL) (85), ALL (82), and CML (86, 87). Disease severity and survival have been shown to be influenced by HIF-1alpha levels, in AML and myelodysplastic syndromes (84, 88, 89); the protein expression of HIF-2alpha, usually absent in normal cells, has been observed in both AML and ALL, but has not been correlated with outcome (82, 90, 91). Thus, leukemic subtype, disease stage or the molecular abnormality involved might explain the variability. To explore potential functions of HIFs in leukemogenesis, various mouse models have been proposed (Table 1). Several studies have shown that inhibition of HIF-1alpha, either by RNAi targeting or by small molecular inhibition, resulted in a failure of primary cells to form *in vitro* colonies and decreased tumor growth and leukemic progression. *In vivo*, dramatic decrease and potential eradication of primary AML cell xenografts have been shown and a complete absence of leukemic induction in secondary transplantation has been reported in cells in which HIF-1alpha was inhibited (79, 84, 85, 92). This has also been confirmed in ALL (93, 94) and CML (83, 87). Similarly, knockdown of HIF-2alpha with shRNA triggers leukemic inhibition (82, 85, 90), which is demonstrated *in vitro* by limited cellular proliferation as well as *in vivo* by absence or poor engraftment.

**Table 1:** **Models used to characterize *HIF1A* and *HIF2A* as oncogenes in leukemias**.

**Leukemia**	Cell types	Mouse models	HIFs inhibition or overexpression	Phenotypes	Reference
**CML**	**Human cells**				
	K562	Nude (in subcutaneous)	Human *HIF1A* cDNA + l-ascorbic acid^a^	No decreased tumor growth	(83)
	**Mouse cells**				
	*Vav-Cre-HIF1a^flox/flox^* transduced with BCR–ABL retrovirus	C57BL/6 J	*Vav-Cre-Hif1a^flox/flox^* system	No leukemia induction in second transplantationDecreased LSCs number	(87)

**AML**	**Human cells**				
	Primary	NOD-SCID	Echinomycin^b^	Decreased/eliminated leukemia cells	(84)
				No leukemia induction in serial transplantation	
	Primary	NOD-SCID gamma	sh*HIF2A*	Decreased/no engraftment	(90)
	HL-60	NOD-SCID gamma	sh*HIF2A*	Increased mice survival	(82)
	NB4	NOD-SCID gamma	sh*HIF1A*, sh*HIF2A*, or EZN-2968^c^	Increased mice survival	(85)
				Delayed leukemia progression	
	SKNO-1	Athymic nude	Echinomycin^b^	Decreased tumor growth	(79)
	HL-60	Nude (in subcutaneous)	l-ascorbic acid^a^	Decreased tumor growth	(83)
	**Mouse cells**				
	PML-RARα Lin^−^	129Sv	*Ex vivo* electroporation with EZN-2968^c^	Increased mice survival	(85)
	PML-RARα Lin^−^	129Sv	EZN-2208^c^	Increased mice survival	(95)
			ATRA^d^ + EZN-2208^c^	Synergy of both treatments to leukemia eradication	
				No leukemia induction in serial transplantation	
	FDCP1	DBA/2	Human *HIF2A* cDNA	Accelerated leukemia progression	(82)
	Relapsed *Mll*^PTD/WT^:*Flt3*^ITD/WT^	C57BL/6 Ly5.1	Echinomycin^b^	Decreased AML blasts (≤20%)	(92)
				Increased mice survival	
	A/E9a transgenic mouse cells	C57BL/6 J	Human *HIF1A* cDNA	Enhanced leukemia disease	(79)
			si*HIF1A*	Suppressed leukemia disease	

**ALL**	**Human cells**				
	Jurkat	Transwell matrigel-coated chambers	Hypoxia (2% O_2_)	Increased tumor invasion	(94)
	Sup-T1				
	Primary T-ALL	NOD-SCID gamma	sh*HIF1A*	Increased mice survival	(93)
				Decreased LSCs frequency	
	**Mouse cells**				
	Primary (NOTCH1-ΔE/NGFR retrovirus)	NOD-SCID gamma	*Hif1a^loxP/loxP^*/CreERT2/GFP system	Increased mice survival	(93)

*^a^l-ascorbic acid indirectly inhibits HIF-1alpha expression by inhibiting NF-κB translocation into the nucleus (83)*.

*^b^Echinomycin binds the core-binding site of HIF-1alpha and inhibits its DNA-binding activity (96)*.

*^c^EZN-2968 and EZN-2208 specifically target HIF-1alpha (85, 95, 97)*.

*^d^ATRA target RARα moiety in PML-RARα mutation of APL (95)*.

Hypoxia *via* HIFs may promote disease maintenance and progression through different mechanisms including energy metabolism (98–100), cycle and quiescence (101, 102), and immune function (103) that are important in normal physiology and deregulated in cancer (47, 104). On one hand, HIF-1alpha and HIF-2alpha influence signaling pathways relevant to leukemia maintenance and propagation. HIF-1alpha activates the Notch1 pathway, which leads to leukemia invasion (94), and promotes the Wnt pathway, consequently preserving LSCs (93). On the other hand, HIF-1alpha acts as an inhibitor of the expression of tumor suppressor genes, such as p15, p16, p19, and p53 (79, 87). Indeed, HIF-1alpha-transactivated DNMT3a methylates DNA, which inhibits tumor suppressors and leads to tumor growth (79). In AML, DNMT3a plays a crucial role since more than 20% of patients exhibit *DNMT3A* mutation (105), conferring a global hypomethylation of DNA and predisposition to developing hematological diseases (106). In contrast, in T-ALL this mutation confers hypermethylation, so the contribution of hypomethylation and hypermethylation to disease development remains to be elucidated (106). Interestingly, taken from non-hematopoietic tissue and cancers, studies have explored the role of hypoxia in epigenetic modifications, through HIF-1alpha stabilization, such as DNA methylation, histone modifications, and non-coding RNA expression (107). Promoter methylation is modified by hypoxia and regulates neural progenitor cell fate (108). The histone demethylase JMJD1A and 1B are targets of HIFs in normal and cancer cells (109–111). Finally in HIF-2alpha-deficient cells, transcriptomic approaches have identified deregulated genes involved in energetic and oxidative metabolisms, plus endoplasmic reticulum (ER) stress, indicating that HIF-2alpha protects AML cells from apoptosis induced by ER stress (90).

One consequences of the expression of HIFs is the promotion of quiescence, which favors chemoresistance. Hypoxia-induced HIF-1alpha promotes entry into G_0_/G_1_ and decreases S phase in AML cells through, in part, upregulation of p27 (112). Quiescence enhances chemoresistance of leukemic cells to cytosine arabinoside (Ara-C) (112, 113) and adriamycin (ADR) (88), since these agents target cycling cells. Coculture of primary AML cells with stromal cells in hypoxia (3% O_2_) confers resistance to Ara-C through stabilization of HIFs and induction of quiescence (28). Antiapoptotic signaling is observed through increased XIAP level, an apoptosis-inhibitory protein, and the activation of the PI3K/AKT pro-survival pathway (112). HIF-1alpha activation by a PHD inhibitor, cobalt chloride (CoCl_2_), protects HL-60 leukemic cells against arsenic trioxide (ATO) by inhibiting BAX and Caspase 3 and 9 and promoting HSP70 protein and p38/ERK pro-survival factors (114). In T-ALL, through Notch1 activation, HIF-1alpha induces BCL2 and BCL-XL upregulation and the downregulation of Caspase 3 and 9 activities, which decreased dexamethasone-induced apoptosis in leukemic cells (94).

Conversely, low oxygen and hypoxia-mimicking agents such as CoCl_2_ or desferrioxamine induce AML cell differentiation through HIF-1alpha accumulation (115, 116). In fact, HIF-1alpha mediates differentiation by binding to C/EBPα and promoting its transcriptional activity (115–117) as well as that of RUNX1 and PU.1 (118, 119). Additionally, C/EBPα/HIF-1alpha induces AML differentiation through c-MYC inhibition and further suppression of miR17 and miR20a expression. The knockdown of p21 and STAT3, two inhibitory targets of miR17 and miR20A, reverses HIF-1alpha-induced AML differentiation (120). In renal cell carcinoma, HIF-1alpha inhibits c-MYC/MAX association, which decreases *c-MYC* promoter binding and thus blocks cells in G_1_ (121). Conversely, HIF-2alpha triggers cell cycle progression and proliferation by enhancing the formation of c-MYC/MAX and its activity. Since HIF-2alpha and HIF-1alpha have dual effects on cell cycle progression according to cell types, more investigations are needed on their antagonistic effects in leukemias. Nguyen-Khac et al. (122) discovered a translocation involving TEL and ARNT in an AML patient exerting a dominant/negative activity on HIF-1alpha. The fusion protein blocks leukemic differentiation, thus conferring a tumor suppressor function for HIF-1alpha. In line with this, data have previously shown that intermittent hypoxia slows down leukemic development in mice (123). However, *in vivo* hypoxia may have unrelated consequences on leukemic cell physiology. More recently, Velasco-Hernandez et al. (124) using different AML models found reduced survival of mice transplanted with HIF-1alpha KO cells (Table 2). These observations were confirmed in myeloproliferative neoplasia through a FLT3^ITD^-induced mouse model (125). Overall, *Hifa* KO enhanced disease progression and severity, making it a tumor suppressor gene. However, authors show that HIF-1alpha deletion may promote compensatory effects *via* overexpression of HIF-2alpha, which may eventually mask the role of HIF-1alpha. This elevation was already seen in HIF-1alpha-deficient cells (90). The *Hif2a* KO in MLL-AF9-driven and Meis1/HoxA9-induced murine AML enhances LSCs development but, once leukemias are established, HIF-2alpha has no impact on their maintenance and propagation. Furthermore, double inhibition of HIF-1alpha and HIF-2alpha demonstrated that HIFs synergize to inhibit AML development, without any role in leukemic propagation (126). Transcriptomic analysis reveals that HIF-1alpha and HIF-2alpha promote a set of genes that fosters survival and proliferation of leukemic cells.

**Table 2:** **Models used to characterize *HIF1A* and *HIF2A* as tumor suppressor genes in leukemias**.

Leukemia	Cell types	Mouse models	HIFs inhibition or overexpression	Phenotypes	Reference
**CML**	**Human**				
	K562	–	Mixture with 10% O_2_ for 5 or 22 + 1 or 24 h of reoxygenation	Survival signals with 5 h hypoxia Death signals with 22 h hypoxia	(127)

**AML**	**Human**				
	MLL-AF9 THP-1 transduced with *HIF2A* sgRNAs + Cas9 nuclease	–	Two independent sgRNAs against the exon 12 of *HIF2A*	Same leukemic propagation than control	(126)
	**Mouse**				
	hMRP8-PML-RARα	FVB-NICO	Intermittent hypoxia equivalent to an altitude of 6000 m (≈10% O_2_) for 18 h/day	Increased mice survival Decreased tumor infiltration/invasion	
	*Hif1a^fl/fl^*; *Mx1-Cre* transduced with *A/E9a* retrovirus	B6SJL	*Hif1a^fl/fl^*/CreERT2 system	HIF-1alpha does not affect AML initiation/progression	(123)
				Decreased mice survival in second transplantation	
	*Hif1a^fl/fl^*; *Mx1-Cre* transduced with *HoxA9-Meis1* retrovirus	C57BL/6 Ly5.1/Ly5.2	*Hif2a^fl/fl^*; *Vav-iCre* system	HIF-1alpha does not affect AML initiation/progression	(124)
	*Hif1a^fl/fl^*; *Mx1-Cre* transduced with *MLL-AF9* retrovirus			HIF-1alpha does not affect AML initiation/progression	
	*Hif2a^fl/fl^*; *Vav-iCre* transduced with *Meis1* and *HoxA9* retroviruses			Decreased mice survival	
				Same AML aggressiveness to control mice	
	*MLL-AF9^KI/^*^+^; *Hif2a^fl/fl^*; *Vav-iCre*	C57BL/6 Ly5.1/Ly5.2	*Hif1a^fl/fl^*; *Hif2a^fl/fl^*; *Vav-iCre* system	Accelerated leukemia disease	(126)
	*Hif1a^fl/fl^*; *Hif2a^fl/fl^*; *Vav-iCre* transduced with *Meis1* and *HoxA9* retrovirus			Decreased mice survival	

Off-target effects of shRNAs and poor specificity of drugs that inhibit HIFs compared to KO might explain this controversy between studies. However, KO may also induce slow cellular adaptation with compensatory effects. Differences may also arise from the model used, mouse versus human, and the different protumoral gene constructions used to generate the leukemia. It will be pertinent to assess the overexpression of HIFs in AML models, and thus observe whether increased HIF delays disease development and, even, eradicate leukemia. HIFs may also differently impact on LSCs and more mature blasts cells, conferring pro-survival effect on LSCs and differentiation on blasts. In favor of this hypothesis, intermittent hypoxia increases survival of APL mice (123). Similarly, in CML, HIF-1alpha induction following short (5 h) hypoxia exposure delivered a survival signal to cells, whereas it promoted cell death within a longer period (22 h) (127). In the ALL model, 24 h-exposure to hypoxia conferred chemoresistance in contrast to longer exposure (48–72 h) (128). These data suggest that duration of hypoxia incubation may promote or inhibit leukemia progression and maintenance, thus explaining the oncogenic or tumor suppressor activity. The link between HIFs and tumor suppressor activity has been previously demonstrated in other cancers (71, 129–131). Taken together, these data suggest that not only the level but also the duration of activity dictates HIF action and hence cellular response in leukemia.

## Therapeutic Strategies: Targeting HIFs or Not?

In light of the results summarized above, it could be difficult to affirm that downregulation of HIFs could be a therapeutic approach. Nevertheless, chemical inhibitors have been tested and the proof-of-concept was first illustrated using echinomycin, which blocks HIF-1alpha-binding activity. This drug preferentially targets AML LSCs through apoptosis, decreasing leukemia burden, prolonging mouse survival, and abrogating disease development in secondary transplantation (84, 92). Echinomycin does not impact on self-renewal and differentiation of normal HSCs, which makes it an ideal molecule to treat leukemia (92). l-ascorbic acid has also been shown to inhibit expression of HIF-1alpha in CML cells and consequently reduces tumor growth. This effect is specific to HIF-1alpha since its overexpression in l-ascorbic acid-treated mice antagonizes leukemic growth inhibition (83). In APL, EZN-2968 and EZN-2208 confer antileukemic activity and prolong mouse survival; in combination with all-trans retinoic acid (ATRA), leukemia eradication was observed, along with survival of mice; fortunately, both compounds are non-toxic to normal HSCs (85, 95). Overall, these data offer new therapeutic options, targeting HIF in leukemia with no impact on normal hematopoiesis. Another approach will be the combination of HIF inhibitors with treatments capable of determining the departure of LSCs from quiescence, and then with treatments that target cycling cells, such as Ara-C.

An alternative strategy will consist of taking advantage of hypoxia to activate drugs and thus to target LSCs in the niche. TH-302 is a hypoxia-activated prodrug, which exhibits a specific cytotoxicity in hypoxia (132, 133). In primary AML, TH-302 hampers tumor growth through multiple mechanisms (cycle arrest, DNA cross-linking, DNA damage). In mouse models, it decreases leukemia burden and prolongs survival (133). PR-104 quickly undergoes alcohol hydrolysis and induces DNA cross-linking in hypoxic cells, impairs ALL progression, decreases tissue infiltration, and prolongs mice survival (134, 135). In a phase I/II study, PR-104 reduced the number of AML and ALL cells in refractory and relapsed patients (136). Despite some side effects, including myelosuppression, febrile neutropenia, and infections, collectively, these results propose innovative therapies for leukemia based on hypoxia-activated prodrugs.

## Conclusion

Overall, these data argue that hypoxia and HIF-mediated signaling play a crucial role in leukemia and leukemogenic processes. However, they conflict in determining whether HIFs act as oncogenes or tumor suppressors, certainly because of the different leukemic models, study design, oxygen level, and hypoxia duration. However, therapies targeting hypoxia and HIFs have proven their efficacy in treating mouse models and may benefit leukemic patients.

## Author Contributions

All authors designed, wrote, and revised the manuscript.

## Conflict of Interest Statement

The authors declare that the research was conducted in the absence of any commercial or financial relationships that could be construed as a potential conflict of interest.
